# Potentiating antilymphoma efficacy of chemotherapy using a liposome for integration of CD20 targeting, ultra-violet irradiation polymerizing, and controlled drug delivery

**DOI:** 10.1186/1556-276X-9-447

**Published:** 2014-08-28

**Authors:** Cong Wu, Huafei Li, He Zhao, Weiwei Zhang, Yan Chen, Zhanyi Yue, Qiong Lu, Yuxiang Wan, Xiaoyu Tian, Anmei Deng

**Affiliations:** 1 Department of Laboratory Diagnosis, Changhai Hospital affiliated to the Second Military Medical University, 168 Changhai Road, Shanghai 200433, China; 2 International Joint Cancer Institute, the Second Military Medical University, 800 Xiangyin Road, Shanghai 200433, China; 3 Institute of Pediatric Research, Children's Hospital affiliated to Soochow University, 303 Jingde Road, Suzhou 215000, China

**Keywords:** Non-Hodgkin lymphoma, Rituximab, Chemotherapy, Liposomes, Serum stability, Ultra-violet irradiation polymerizing

## Abstract

Unlike most malignancies, chemotherapy but not surgery plays the most important role in treating non-Hodgkin lymphoma (NHL). Currently, liposomes have been widely used to encapsulate chemotherapeutic drugs in treating solid tumors. However, higher *in vivo* stability owns a much more important position for excellent antitumor efficacy in treating hematological malignancies. In this study, we finely fabricated a rituximab Fab fragment-decorated liposome based on 1,2-bis(10,12-tricosadiynoyl)-sn-glycero-3-phosphocholine (DC8,9PC), which can form intermolecular cross-linking through the diacetylenic group by ultra-violet (UV) irradiation. Our experimental results demonstrated that after the UV irradiation, the liposomes exhibit better serum stability and slower drug release with a decreased mean diameter of approximately 285 nm. The cellular uptake of adriamycin (ADR) by this Fab-navigated liposome was about four times of free drugs. Cytotoxicity assays against CD20^+^ lymphoma cells showed that the half maximal (50%) inhibitory concentration (IC50) of ADR-loaded immunoliposome was only one fourth of free ADR at the same condition. *In vivo* studies were evaluated in lymphoma-bearing SCID mice. With the high serum stability, finely regulated structure, active targeting strategy via antigen-antibody reaction and passive targeting strategy via enhanced permeability and retention (EPR) effect, our liposome exhibits durable and potent antitumor activities both in the disseminated and localized human NHL xeno-transplant models.

## Background

Non-Hodgkin lymphoma (NHL) is a type of blood cancer, which presents not only as a solid tumor of lymphoid cells in lymph nodes and/or extranodal lymphatic organs such as spleen and bone marrow, but also as free lymphoma cells in circulating blood [1-3]. Particularly, most patients can be cured with chemotherapy and/or radiation, which revealed the important status of chemotherapy in the treatment of NHL [4-6]. Currently, while various chemotherapeutic agents are validated to be effective in the treatment of lymphoma in preclinical studies, clinical applications are often limited for their side effects to normal tissues because of the systemic administration. As a result, finding more effective strategy to maximize the curative effect while minimizing the side effects of chemotherapy against lymphoma is of great importance and urgency [7,8].

In the past decade, nanocarriers, including liposomes, polymeric nanoparticles, micelles, nanogels etc., with an appropriate diameter of tens to hundreds of nanometers, have received widespread attention for the specific delivery of bioactive reagents in the diagnosis and treatment of cancer [7,9-12]. Encapsulation of bioactive reagents in nanocarriers can result in significant accumulation and retention in solid tumor tissues relative to administration of drug in conventional formulations through the enhanced permeability and retention (EPR) effect, which was firstly described by *Maeda and colleagues*[13-17]. What's more, the drug loading nanocarriers owns high serum stability, which can contribute to long-time circulation in the blood vessels, resulting in long-lasting antitumor activities, especially for the killing of free malignant cells in circulating blood [12,17,18]. However, more and more laboratory researches and clinical studies have demonstrated that passive targeting strategy alone is not enough for more sufficient and efficient accumulation of drug-loading nanocarriers in some tumor types [19-21].

Following the FDA approval of anti-CD20 mAb Rituximab for CD20^+^ NHL treatment, monoclonal antibody (mAb)-based targeting therapy has revolutionized the treatment of malignancies for the specific antitumor activity and low cytotoxicity against normal tissues [22,23]. In the last decade, more and more studies have confirmed that the combination of mAb-based active targeting and nanoparticle-based passive targeting can improve drug concentration in tumor tissues and tumor cells in a shorter time with greater accuracy [7,24,25].

In this study, we have developed an adriamycin (ADR)-loaded liposome using the diacetylenic phosphatidylcholine 1,2-bis(10,12-tricosadiynoyl)-sn-glycero-3-phosphocholine (DC8,9PC, hereafter referred to as PC), which can form intermolecular cross-linking through the diacetylenic group to produce a conjugated polymer within the hydrocarbon region of the bilayer by ultra-violet (UV) irradiation (Additional file 1: Figure S1) [26,27]. For the sake of active targeting, the Fab fragments of rituximab were conjugated onto the liposomal surface. Our experimental results demonstrate that this well-modified liposome, which owns good serum stability and prolonged circulation time, can accumulate in the tumor tissues and malignant cells with high specificity and sufficient amount, which can bring out exceptional excellent and durable therapeutic efficacy against CD20-positive lymphomas.

## Methods

### Cell lines and materials

Two human B cell lymphoma cell lines, Raji and Daudi, were obtained from the American Type Culture Collection (ATCC). Cells were propagated and maintained in RPMI 1640 supplemented with 10% (*v*/*v*) fetal bovine serum (FBS, GIBCO, Invitrogen, Carlsbad, CA, USA) in a controlled atmosphere incubator at 37°C with 5% CO_2_. The DC8,9PC and 1,2-distearoyl-sn-glycero-3-phosphoethanolamine-*N*-[maleimide(polyethylene glycol)-2000] (Mal-PEG) were purchased from Avanti Polar Lipids (Williamsport, PA, USA). The anti-CD20 antibodies rituximab was purchased from Roche (Basel, Switzerland).

### Fabrication of Fab fragment-conjugated liposome (Figure 1)

**Figure 1:**
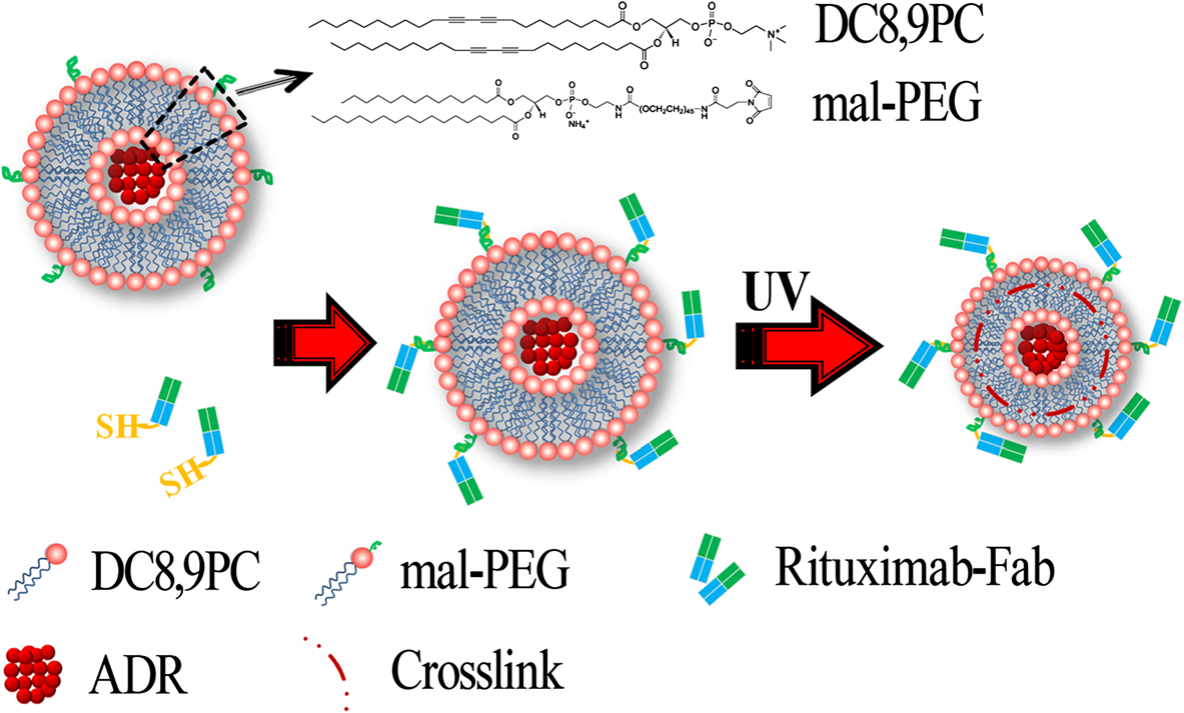
Fabrication of rituximab Fab fragment-decorated liposomes.

#### *Formation of drug-loaded liposomes*

Total lipids mixtures of 2 mg DC8,9PC and 0.25 mg Mal-PEG were dissolved in 500 μL mixed solvent of chloroform and methyl alcohol with the volume ratio at 1:1. Then, the solvent was evaporated under vortex and flashed with nitrogen to obtain the lipid film, followed by washing-out with 2 mL of ADR (doxorubicin HCl, Melonepharma CO. LTD., Dalian, China) solution (0.5 mg/mL in PBS) to obtain ADR-loaded multilamellar vesicles [26]. The collected liposome solution was dialyzed against PBS using a membrane (molecular weight cutoff 3 kDa) at 4°C for 12 h to remove uncombined ADR resulting in the ADR-loaded liposome stocking solutions.

#### *Thiolation of mAbs*

The Fab fragment of rituximab was prepared as reported previously [25]. Briefly, 10 mg/mL Rituximab was incubated with 0.25 mg/mL de pepsin at 37°C overnight following with a centrifugation and dialysis against TBS (145 mM NaCl and 10 mM Tris, pH 7.5) for 18 h. The Staph A-Sepharose column (Pharmacia, Kalamazoo, MI, USA) was used to remove the undigested mAbs at pH 8.0 resulting in the rituximab F(ab)_2_. The obtained F(ab)_2_ was further purified by the Sephadex G-150 column (Pharmacia, MI, USA) which was pre-equilibrated by the buffer 1 (0.1 M NaCl, 0.1 M borate, 0.05 M citrate and 2 mM EDTA, pH 5.5). Such F(ab)_2_ solution was concentrated to 10 mg/mL and further digested by the enzyme papain. The Fab fragment solution was purified by the same procedure as mentioned above resulting in the single Fab fragment stocking solution storing at 4°C.

To activate the Fab fragments of rituximab for reactivity toward the maleimide, the above stocking solutions were incubated with 2-iminothiolane (2-IT, Sigma-Aldrich, St. Louis, MO, USA) with a mass ratio of 1:0.15 (Fab/2-IT) at room temperature for 2 h under a gentle shake. Unreacted 2-IT was removed by dialysis. The bovine serum albumin (BSA) ~ SH was produced in the same way. The resulting reactive Fab ~ SH and BSA ~ SH were stored at 4°C for future usage [28].

#### *Fabrication of rituximab Fab-conjugated liposome*

The Fab fragment-conjugated liposome was prepared by coupling the reactive Fab ~ SH onto the liposomal surface via the reaction between the ~ SH and Mal-group at 4°C and N_2_ environment overnight; the un-conjugated Fabs were removed by dialysis. The BSA-conjugated liposome was fabricated in the same way. For UV irradiation, pure liposome solutions were exposed to 20 irradiation cycles at 4°C, with a 254-nm UV light dose of 360 mJ/cm^2^ per cycle using a Stratalinker-UV 1800 [26]. The concentration of Fab fragments in the liposome solution was quantified by measuring the A260/A280 using Nano VueTM (GE Healthcare, Wilmington, MA, USA).

### Characterization of Fab fragment-conjugated liposome

The hydrodynamic diameter and size distribution were determined by ZetaSizer (Nano-ZS, Malvern Instruments, Worcestershire, UK) equipped with a HeeNe laser (633 nm) at the scattering angle 173°. To prepare stained specimens for TEM (H-7000 Electron Microscope, Hitachi, Tokyo, Japan) experiments, about 5 μL liposome solution was dropped on 200-mesh Formvar-free carbon-coated copper grids (Ted Pella Type-A; nominal carbon thickness 2 to 3 nm). After the water evaporating by exposing to air at room temperature, the sample was inversely covered on a small drop of hydrodated phosphotungstate (PTA) solution with a mass fraction of 2%. The conventional TEM images were obtained at 100 kV.

### Weight-average molecular weight analysis by SLS

The static light scattering (SLS) measurements were carried out varying the scattering angle (θ) from 40 to 140° with a 5° stepwise increase [29]. The weight-average molecular weight (*M*_*w*_) was estimated from the following equation [30]:

KCPRq=1Mw+2A2C

where *K* = [4*π*^2^*n*^2^(*dn*/*dC*_*P*_)^2^]/*N*_AV_*λ*^4^ is optical contrast, with *n* being the refractive index of solvent, *C*_*p*_ the liposomal concentration, *dn/dC*_*p*_ the refractive index increment against *C*_*p*_ determined by a double beam differential refraction meter (DMR-1021) (Otsuka Electronics, Tokyo, Japan), *λ* the incident wavelength, and *N*_AV_ the Avogadro's number. *R*(*q*) is the Rayleigh ratio at a specific measurement angle. By measuring *R*(*q*) for a set of *θ* and *C*_*p*_, values of *M*_*w*_ and *A*_2_ were estimated from typical Zimm plots.

### ADR releasing profile

A dialysis bag (molecular weight cutoff 1 kDa) containing 3 mL PC-ADR solution before or after UV irradiation was respectively put in a beaker with 500 mL PBS. The beaker was fixed in a water bath kept at 37°C with continues siring. About 500 μL PBS solution outside the dialysis bag was sampled at different time intervals, which was measured by UV at 480 nm to determine the ADR concentration. The cumulative drug release was calculated by the following function:

$$\text{Cumulative}\;\text{release}=\frac{C_{\text{Outside}\;\text{dialysis}\;\text{bag}}\times 500\;\text{mL}}{C_{\text{Inside}\;\text{dialysis}\;\text{bag}}\times 3\;\text{mL}}\;\times 100\%$$

### Serum stability evaluation by DLS

For evaluating the effect of UV irradiation on the liposomal stability, a bovine serum albumin (BSA) solution in RPMI 1640 with a concentration of 50% (*m*/*v*) was used as an *in vitro* serum model to mimic the *in vivo* status. Then, the irradiation (irrad) and non-irrad liposome solutions were separately mixed with the resulting serum model at 37°C for 24 h. The dynamic light scattering (DLS) was used to measure the size and size distribution profile of BSA/liposome mixture at 0 and 24 h, respectively.

### Cellular uptake and internalization assays

Raji and Daudi cells were seeded into a 48-well microplate (1 × 10^5^ cells) and incubated with 1 μg/mL free ADR, ADR-loaded liposomes decorated with Fab fragments (PC-ADR-Fab), or BSA (PC-ADR-BSA) in cell culture medium containing 1% (*v*/*v*) antibiotics at 37°C for 4 h. Cells incubated with culture medium were used as a negative control. After washing with PBS for twice, a FACScan Flow Cytometer (Becton Dickinson, San Jose, CA, USA) was used to assess the cellular uptake of ADR or ADR-loaded liposomes by detecting the mean fluorescence intensity (MFI) of FL-2 (ADR fluorescence). Additionally, each sample was also visualized using an inverse fluorescent microscopy.

### *In vitro* cytotoxicity assay

Cytotoxicity assessment was carried out on Raji and Daudi cells using a Cell Counting Kit-8 (CCK-8, Beyotime Institute of Biotechnology, Shanghai, China) assay. Briefly, cells were seeded in a 96-well plate at an initial density of 3,000 cells/well in 100 μL of RPMI-1640 supplemented with 10% (*v*/*v*) heat-inactivated FBS, 1% (*v*/*v*) antibiotics, and different concentrations of free ADR, PC-ADR-BSA, or PC-ADR-Fab or the corresponding concentration of rituximab Fab. After 48 h, 10 μL CCK-8 was added to each well for another 2-h incubation protected from light. The absorbance (Ab) at 450 nm was recorded by a micro-plate reader (Thermo Multiskan MK3, Thermo Scientific, Waltham, MA, USA), and cell viability was calculated as the following function:

$$\text{Cell}\;\text{viability}\%=\frac{\left({\text{Ab}}_{\text{Sample}}-{\text{Ab}}_{\text{Blank}}\right)}{\left({\text{Ab}}_{\text{Control}}-{\text{Ab}}_{\text{Blank}}\right)}\times 100\%$$

where Ab_sample_, Ab_control_, and Ab_blank_ are the absorbance values of each sample, the cells cultured in culture medium without any additional substances, and the culture medium without cells in wells, respectively.

### Animals

Healthy female SCID mice aged about 4 weeks were purchased from Shanghai Experimental Animal Center of Chinese Academic of Sciences (Shanghai, China), housed in specific pathogen-free conditions, and treated in accordance with guidelines of the Committee on Animals of Changhai Hospital affiliated to the Second Military Medical University (Shanghai China).

### Pharmacokinetics and *in vivo* distribution analysis

The pharmacokinetics (PK) and *in vivo* distribution analysis was done following *Joseph M. Tuscano*'s study with minor revisions [31]. Briefly, Daudi cells (1 × 10^7^) were inoculated subcutaneously into the right flank of 6-week-old SCID mice. For PK assays, when tumors reached about 50 to 60 mm^3^ in volume (approximately 14 days), mice were randomly administrated tail vein injection of free ADR, non-irrad or irrad ADR-containing immunoliposomes at a dosage of 5 mg ADR/kg (*n* = 3 mice per treatment). Then, 10 μL of blood were collected through tail vein nicking from each mouse at 5, 15, and 30 min and 1, 2, 4, 6, 8, 12, 24, and 48 h after treatment, respectively. Samples were immediately diluted into 250 μL of 0.5 mmol/L EDTA-PBS, followed by a centrifugation (300 *g* × 5 min). Plasma was collected and ADR was extracted by acidified isopropanol (75 mmol/L hydrochloric acid in 90% isopropanol) at 4°C for 20 h. The ADR concentrations were measured by UV at 480 nm and expressed as micrograms per milliliter (ADR/blood plasma). The data were analyzed by the PK solver software [32]. For biodistribution assays, tumor-bearing mice were randomly administrated tail vein injection of free ADR, PC-ADR-BSA, or PC-ADR-Fab at a dosage of 5 mg ADR/kg (*n* = 3 mice per treatment). Mice were sacrificed 24 h after treatment; part of tumor, heart, liver, spleen, kidneys, and lungs were removed, washed, and weighed; and single-cell suspensions were made. ADR was extracted from cells by the abovementioned acidified isopropanol for 20 h at 4°C. The ADR concentrations were determined as described above and expressed as micrograms per gram (ADR/tissue). What's more, part of the tumor tissues were collected and subjected to frozen sections, which were detected by a confocal microscrope (Zeiss, Oberkochen, Germany).

### *In vivo* antitumor activity assessment in disseminated human NHL xeno-transplant models

Six-week-old SCID mice were injected via the tail vein with 5 × 10^6^ Daudi cells in 100 μL PBS. Then, the inoculated mice were randomly assigned to 4 groups with 10 each for the treatment of PBS, free ADR, PC-ADR-BSA, and PC-ADR-Fab (with an equivalent amount of 5 mg/kg ADR) via the tail vein weekly for 3 times after 48 h. Post-operation monitoring was exercised at least once a day until natural death in a range of 120 days. Survival curves were plotted with the Kaplan-Meier method and compared by using a log-rank test [33,34].

### *In vivo* antitumor activity assessment in localized human NHL xeno-transplant models

Daudi cells (1 × 10^7^) in 100 μL of PBS buffer were inoculated subcutaneously into the lateral flank of 6-week-old SCID mice. When the tumors reached about 50 to 60 mm^3^ in volume, the inoculated mice were randomly assigned to four groups with four each for the treatment of PBS, free ADR, PC-ADR-BSA, and PC-ADR-Fab (with an equivalent amount of 5 mg/kg ADR) via the tail vein weekly for three times. Post-operation monitoring was exercised at least once a day, and the tumor size was measured in two perpendicular diameters with precision calipers every 3 days and calculated in a range of 60 days. Tumor volume was measured according to the following formula [25]:

$$\text{Tumor}\;\text{volume}=\text{Length}\times {\text{Width}}^{2}/2$$

where length and width refers to the longest and the shortest diameters of tumors, respectively.

### Statistical analysis

Data were expressed as the means ± standard deviation (SD). Statistical analysis was performed by Student's *t* test or one way ANOVA to identify significant differences unless otherwise indicated. Differences were considered significant at a *P* value of <0.05.

## Results

### Characterization of the liposome

**Figure 2:**
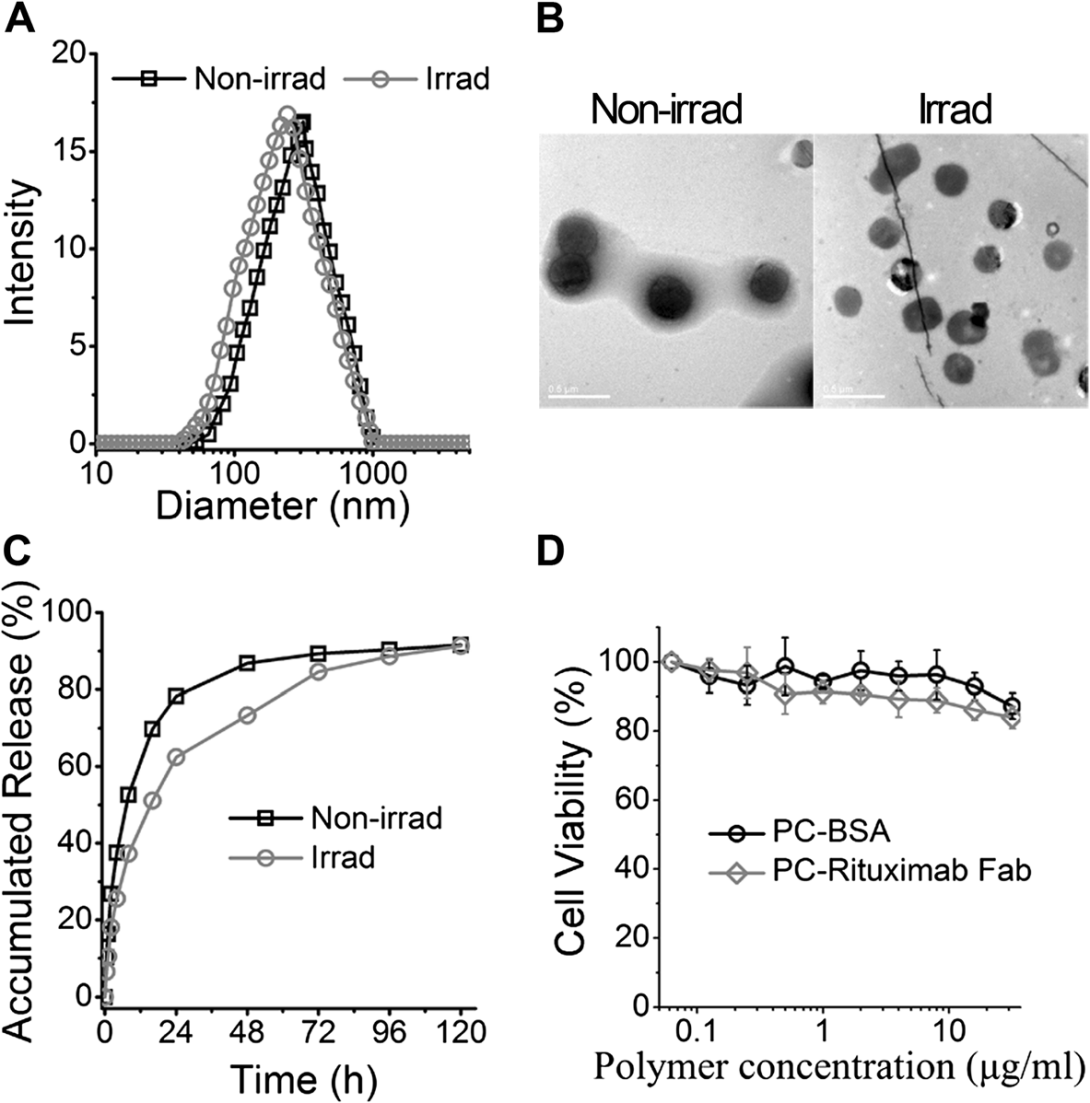
**Properties of CD20 targeting liposomes. (A)** Size distribution of liposomes before or after UV irradiation. **(B)** The TEM morphology of the liposomes before or after UV irradiation, scale bar 0.5 μm. **(C)** The drug release profile of ADR-loaded liposomes before or after UV irradiation. **(D)** The cytotoxicity profile of the empty liposomes PC-BSA and PC-Fab incubated with CD20 overexpressed Raji cells.

It has been firmly established that size distribution of a liposome strongly affect its *in vitro* and *in vivo* performances [17,25]. Therefore, we firstly assessed the size distribution of our liposome after the successful fabrication. Figure 2A shows the size distribution of irrad and non-irrad liposomes. It was illustrated that an 11% decrease in mean size was occurred after UV irradiation (from approximately 321 nm before irradiation to 285 nm after irradiation). This interesting physical change was validated by morphology analysis using a TEM, of which the results suggested that both the irrad and non-irrad liposome showed a regular spherical morphology with different diameters (Figure 2B).

### Fab fragment loading

The number of Fab fragments per liposome was estimated on the basis of *Kozlowska's* ideas according to the following equation [35]: $\text{Fab}\;\text{fragments}\;\text{per}\;\text{liposome}=\frac{\text{Fab}\;\text{molecules}\;\text{per}\;\text{mL}}{\text{liposomes}\;\text{per}\;\text{mL}}\text{.}$

Firstly, the liposomal *M*_*w*_ was estimated to be 1.22 × 10^7^ g/mol by SLS analysis (Table 1), and the Fab concentration in liposome solution was quantified to be 52.2 μg/mL by determining the A260/A280 by Nano VueTM. Besides, the total mass of liposomes in the suspensions (total volume 2.85 mL) can be calculated from the original polymer amount of 2 mg PC and 0.25 mg Mal-PEG plus the detected amount of Fab (52.2 μg/mL × 2.85 mL) to be approximately 2,398.8 μg. The total mass of liposomes per milliliter can be calculated to be 841.7 μg (2,398.8 μg/2.85 mL). Accordingly, we can estimate that there are 6.9 × 10^-11^ mol [841.7 μg/(1.22 × 10^7^ g/mol)] or 4.15 × 10^13^ liposomes per milliliter.

**Table 1:** Physicochemical parameters of ADR-loaded immunoliposomes

***R***_***h ***_**(nm)**	**PDI**	***M***_***w ***_**(g/mol)**	***N***_**agg**_	**Fab/liposome**	**ADR (ng)/liposome**
141.3	0.055	1.22 × 10^7^	1,151	31.3	3.1 × 10^-9^

*R*_*h*_, averaged radius; PDI, particle dispersion index; *M*_*w*_, weight-average molecular weight; *N*_agg_, the liposomal aggregation number; Fab/liposome, Fab fragments per liposome; ADR/liposome, ADR mass per liposome.

The number of Fab fragments (24 kDa) per milliliter calculated in the same way was 2.2 × 10^-9^ mol [52.2 μg/(2.4 × 10^4^ g/mol)] or 1.3 × 10^15^. Hence we can estimate that there are on average ~31.3 Fab fragments per liposome (1.3 × 10^15^ Fab fragments/4.15 × 10^13^ liposomes), which is also shown in Table 1.

### Drug loading and releasing properties

It was well expected that our liposome could be an excellent drug carrier which benefits from the stable structure following by self-assembling and UV irradiation functions. For the validation of this expectation, we firstly evaluated the ADR loading content (LC) of our liposomes according to the following function: $\text{Loading}\;\text{Content}\;\left(\text{LC}\right)=\frac{C_{\text{ADR}}}{C_{\text{polymer}}}\times 100\%$. The results revealed a relative high LC of 16.27% with our immunoliposomes. Besides, the amount of ADR per liposome was estimated to be 3.1 × 10^-9^ ng (Table 1), which was calculated according to the following equation:

$$\text{ADR}/\text{liposome}=\text{ADR}\;\left(\text{ng}\right)\;\text{per}\;\text{mL}/\text{liposomes}\;\text{per}\;\text{mL}$$

Also, the drug release profiles were determined in PBS buffer at a PH value of 7.4 at 37°C. As expected (Figure 2C), slower drug release from the irrad liposomes was observed comparing with non-irrad liposomes. This controlled drug release can be attributed to the polymerization of PC by UV light irradiation. Otherwise, approximately 62%, 73%, 84%, 88%, and 91% of ADR was respectively released from the irrad liposomes after 24, 48, 72, 96, and 120 h, the fact of which ensures sufficient drug release at the tumor site, especially in tumor cells.

### Low cytotoxicity of liposomes

For the determination of the cytotoxicity, different concentrations of empty liposomes decorated by BSA (PC-BSA) and rituximab Fab fragments (PC-Fab) were incubating with Raji cells at 37°C for 48 h following by a CCK-8 detection. As illustrated in Figure 2D, both the PC-BSA and PC-Fab showed low cytotoxicity to Raji cells in concentrations of up to 32 μg/mL. It is worth mentioning that the cell viability of PC-Fab-incubated cells had a little decrease compared with PC-BSA-incubated cells, which may be related with the weak tumor suppression effect of rituximab Fab fragments.

### Serum stability evaluation

**Figure 3:**
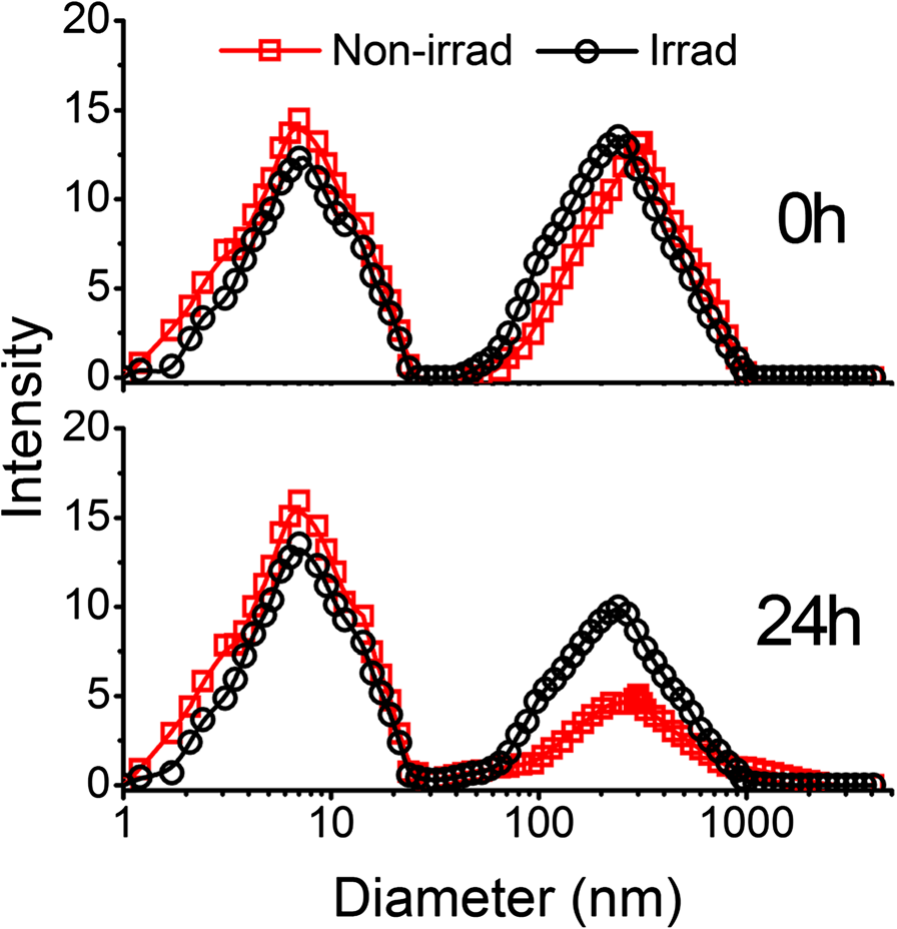
**Liposomal *in vitro *serum stability assessment.** Up panel: size distribution of the liposome dilution in RPMI 1640 containing 50% (*m*/*v*) BSA. Down panel: size distribution of the above dilution after the incubation at 37°C for 24 h. Red, liposomes before UV irradiation; black, liposome after UV irradiation.

For future clinical applications, the *in vivo* stability of liposome is another important factor which should be considered. Therefore, we used the RPMI 1640 containing 50% BSA as an *in vitro* model of serum to check the serum stability profile of our liposomes, in which the existence of BSA was employed to mimic a variety of serum proteins in the complicated environment within the blood vessels. Figure 3 shows the size distribution of liposome solutions before (red) and after (black) UV irradiation. The first individual peak in each histogram represents the size distribution of BSA, and the second represents that of liposomes. The results indicated that after the dilution of liposomes in serum model, the size distribution of each sample was similar as separately measured (Figure 3A), while after a 24-h incubation, the well-separated peaks for BSA and liposomes still appeared in the mixture, which is an indication of good serological stability. However, the non-irrad liposomes in the mixture showed a much broader size distribution (Figure 3B). The results revealed that after the UV irradiation, our liposomes showed better stability in the serum model than non-irrad ones.

### Intracellular uptake of liposomes

**Figure 4:**
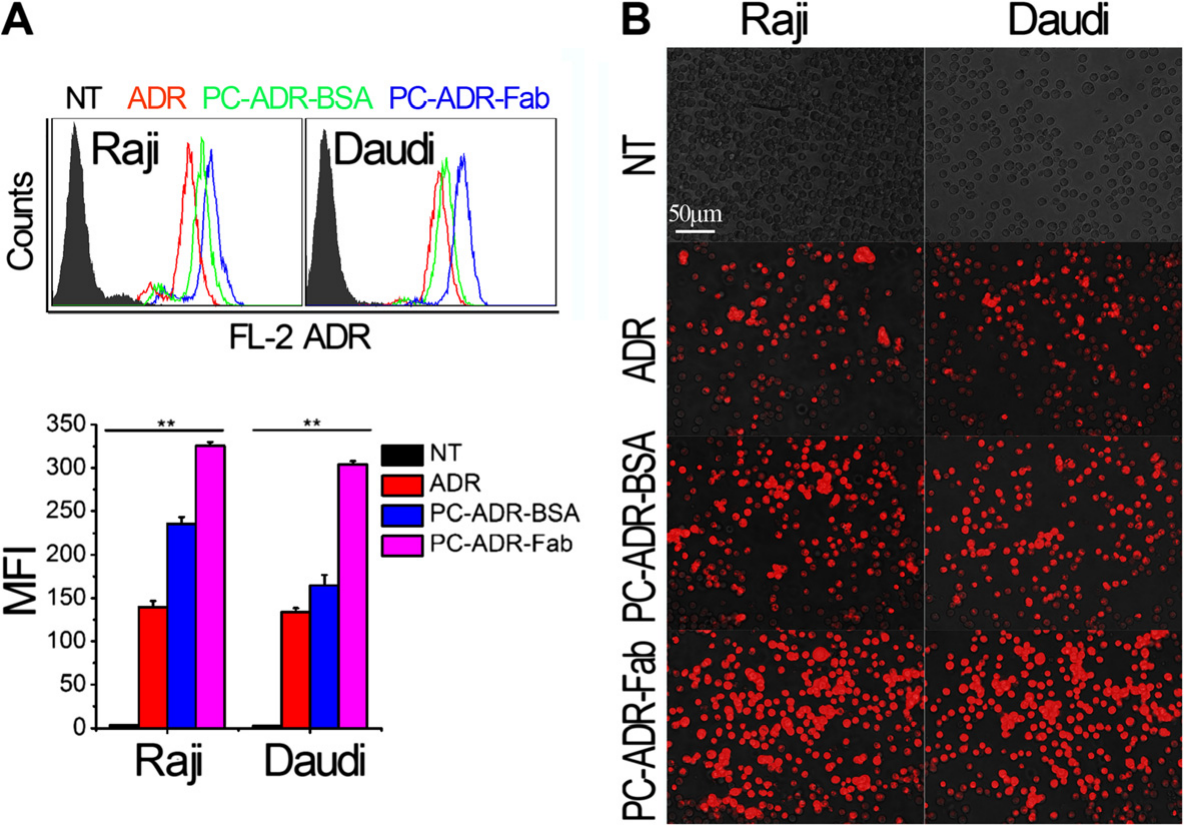
**Cellular uptake and intracellular accumulation of ADR-loaded liposomes. (A)** Detection of ADR fluorescence intensity by FCM. Up panel: the histogram represents the fluorescence intensity distribution of Raji and Daudi cells. Black histogram, no-treat; red histogram, free ADR treatment; green, PC-ADR-BSA treatment; blue, PC-ADR-Fab treatment. Down panel: Numerical data representing the mean fluorescence intensity (MFI) of ADR fluorescence in Raji and Daudi cells. Data are mean ± SD of at least three experiments. **(B)** The effects of liposomes on the intracellular uptake indicated by the inverse fluorescent microscopy. Red fluorescence represents the intracellular ADR. Scale bar 50 μm.

For the evaluation of intracellular uptake of our CD20-targeting liposomes, the ADR-loaded liposomes, PC-ADR-BSA and PC-ADR-Fab, were incubated with CD20^+^ Raji and Daudi cells for 4 h. After washing, the flow cytometer (FCM) and inverse fluorescent microscopy were used to evaluate the ADR fluorescence (red) in lymphoma cells. As indicated by the mean fluorescence intensity (MFI) of FL-2 (Figure 4A), the PC-BSA (green hitograms) and PC-Fab (blue hitograms) significantly enhanced the intracellular uptake of ADR compared with free drugs (red hitograms) (**p = 0.000), while the increasing extent of PC-Fab is much higher than that of PC-BSA (**p = 0.000). This result was confirmed by the inverse fluorescent microscopy as displayed in Figure 4B.

### *In vitro* cytotoxicity assays

**Figure 5:**
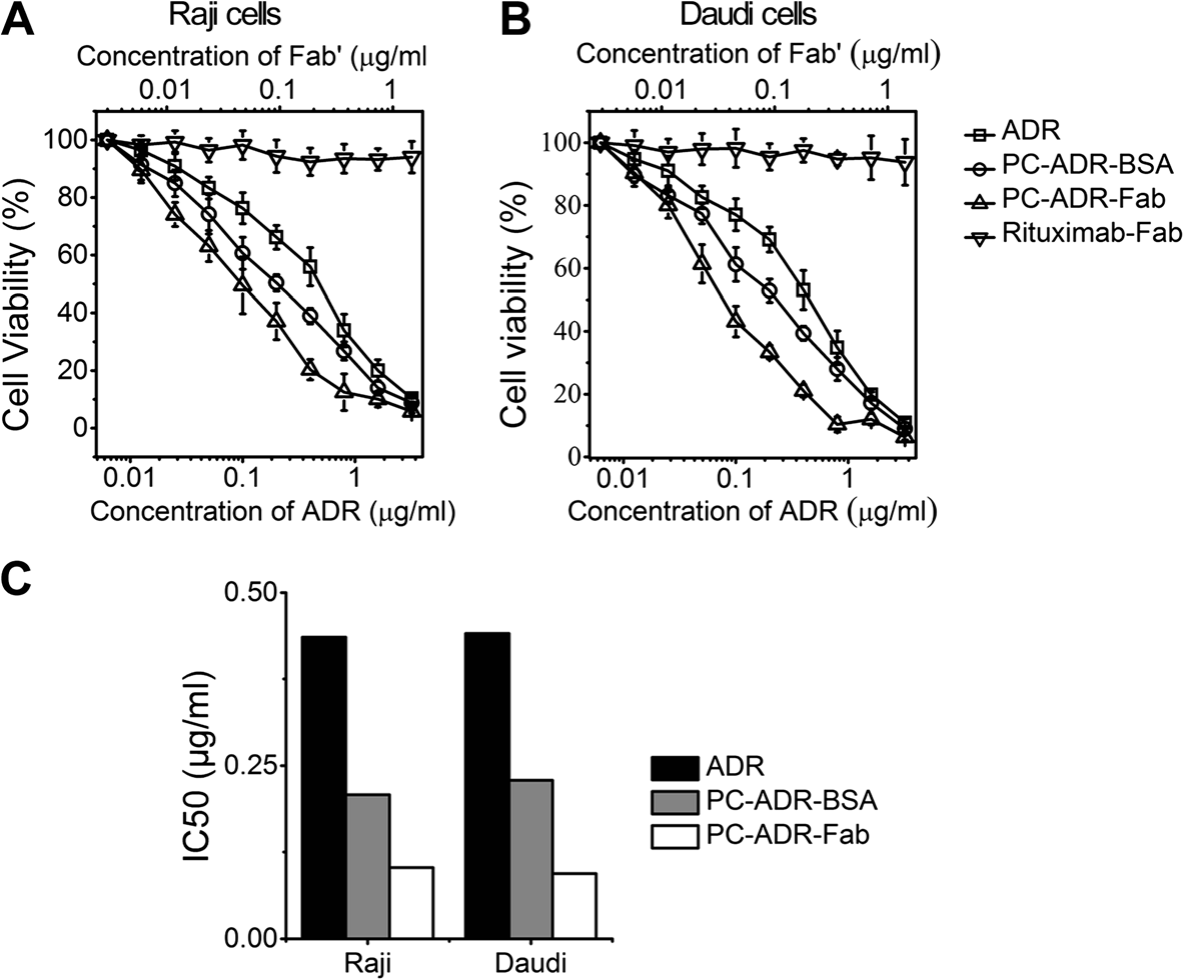
***In vitro *antitumor activity of ADR loaded liposomes.** Concentration-dependent cytotoxicity evaluation of free ADR, rituximab Fab, PC-ADR-BSA, and PC-ADR-Fab in Raji cells **(A)** and Daudi cells **(B)**. **(C)** The IC50 to Raji and Daudi cells of free ADR, PC-ADR-BSA, and PC-ADR-Fab.

The *in vitro* antitumor activities of our liposomes were subsequently evaluated. After the incubation of Raji and Daudi cells with different concentrations of free ADR, rituximab Fab fragments, PC-ADR-BSA, and PC-ADR-Fab for 48 h, a CCK-8 assay was employed to determine the cell viability. As illustrated in Figure 5A,B, both Raji and Daudi cells showed a significantly lower cell viability after the treatment of drug-loaded liposomes compared with free ADR, while PC-ADR-Fab exhibited more potent antitumor activity compared with PC-ADR-BSA in all the tested ADR concentrations. Because the therapeutic effects of rituximab is largely dependent on the Fc-related antibody-dependent cell-mediated cytotoxicity (ADCC) and complement dependent cytotoxicity (CDC) [36], the Fab fragments demonstrated low cytotoxicity in both Raji and Daudi cells in all the tested concentrations (0.005 to 1.3 μg/mL), which corresponded to the ADR concentrations in the liposomal system. Furthermore, the half maximal (50%) inhibitory concentration (IC50) of ADR was calculated to evaluate the cytotoxicity of the liposomal drug delivery systems according to the ADR concentration dependence of the cell viability profile. It was shown in Figure 5C that PC-ADR-Fab demonstrated the lowest IC50 to Raji (0.103 μg/mL) and Daudi (0.094 μg/mL) cells compared with PC-ADR-BSA (IC50_Raji_ 0.208 μg/mL, IC50_Daudi_ 0.229 μg/mL) and free ADR agents (IC50_Raji_ 0.436 μg/mL, IC50_Daudi_ 0.441 μg/mL).

### Pharmacokinetics of ADR-containing liposomes in tumor bearing SCID mice

After a short injection of free ADR and ADR-containing liposomes at 5 mg/kg into lymphoma-bearing SCID mice, the plasma ADR concentrations were measured at different time intervals. The data were analyzed using the PK solver software [32] and the results are all fitted to a trilocular pattern [37]. The time-concentration curve is shown in Additional file 2: Figure S2 and the PK parameters in Table 2. As we can see, a remarkable difference in plasma PK was observed after the tail vein administration of free and liposomal ADR. The *t*_1/2_γ (the elimination half time in the elimination phase) was relatively longer for irrad liposomes (34.53 ± 2.63 h) than that for non-irrad liposomes (21.13 ± 1.50 h) and free drugs (9.56 ± 4.06 h). In contrast, the clearance (CL) was significantly reduced for irrad liposomes (6.63 ± 3.74 ml/h versus CL_non-irrad liposomes_ 8.82 ± 4.54 ml/h, CL_free drugs_ 30.96 ± 5.86 ml/h).

**Table 2:** **Tumor bearing nude mice serum pharmacokinetic parameters comparing free and liposomal ADRs (*n*** **= 3)**

**Parameter**	**Unit**	**Free ADR**	**Non-irrad**	**Irrad**
*t*_1/2_α	h	0.20 ± 0.02	0.19 ± 0.04	0.21 ± 0.05
*t*_1/2_β	h	0.98 ± 0.19	3.89 ± 0.79	1.57 ± 1.31
*t*_1/2_γ	h	9.56 ± 4.06	21.13 ± 1.50	34.53 ± 2.63
CL	mL/h	30.96 ± 5.86	8.82 ± 4.54	6.63 ± 3.74
*C*_max_	μg/mL	50.45 ± 5.54	54.13 ± 4.34	53.04 ± 5.68
AUC_0-t_	(μg/mL) **·** h	79.97 ± 11.36	447.19 ± 54.19	713.49 ± 120.51
MRT	h	6.37 ± 2.15	27.54 ± 1.53	48.58 ± 4.67

*t*_1/2_, the elimination half time; CL, clearance; *C*_max_, the maximum observed concentration in the plasma; AUC, area under the concentration-time curve; MRT, mean residence time.

### *In vivo* distribution and tumor accumulation assays

**Figure 6:**
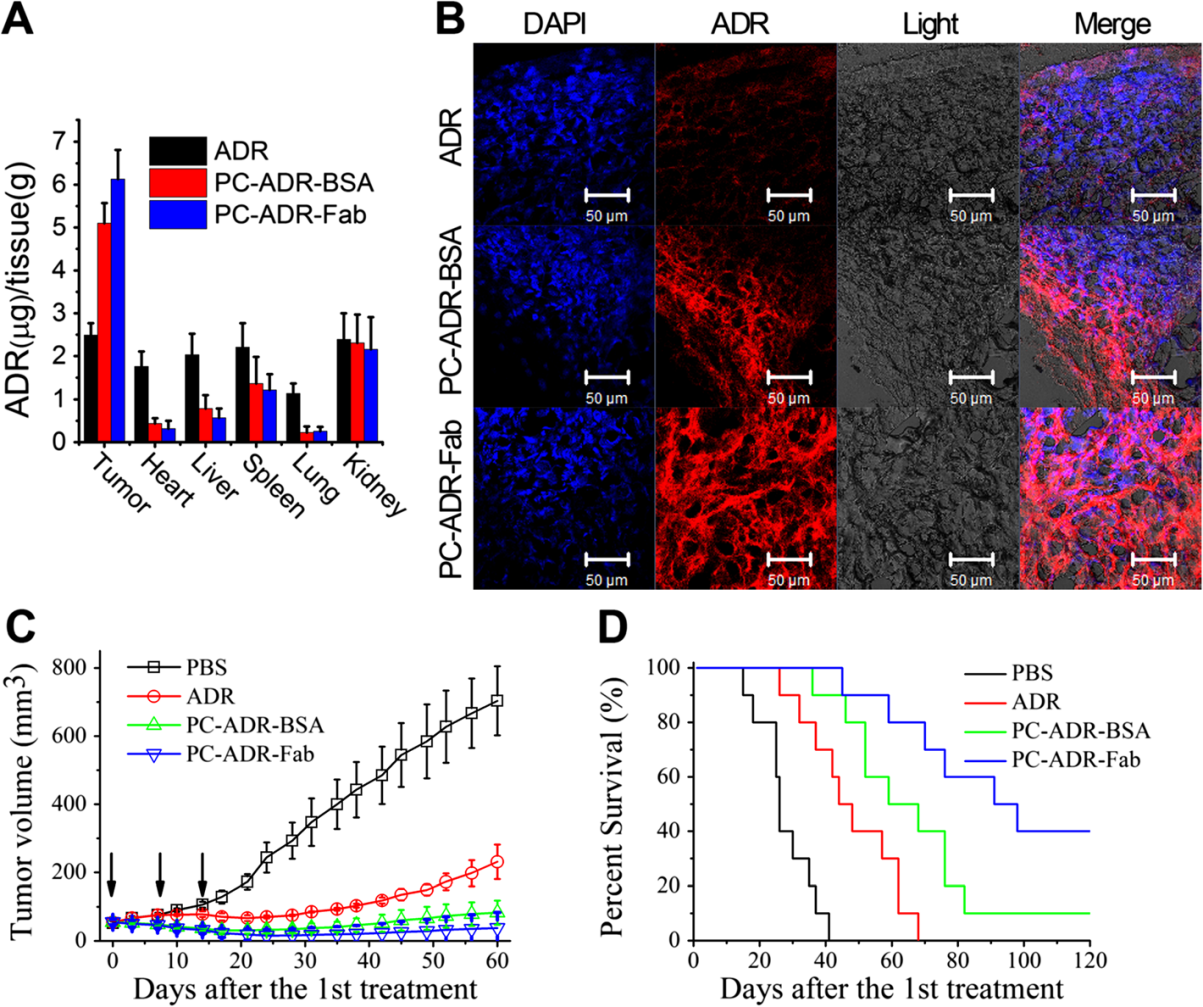
***In vivo *antitumor activity of ADR-loaded liposomes. (A)** Lymphoma-bearing SCID mice were treated with 5 mg/kg free ADR, PC-ADR-Fab, and PC-ADR-Fab; 24 h later, mice were euthanized and organs were harvested, washed, and weighed; and the ADR was extracted and quantified. **(B)***In vivo* tumor accumulation profile of frozen section from lymphoma-bearing SCID mice treated with free ADR, PC-ADR-BSA, and PC-ADR-Fab for 24 h as visualized by confocal microscopy, the RED fluorescence represents the tumor accumulation and retention of ADR. Scale bar 50 μm. **(C)***In vivo* anticancer therapeutic effects in localized human NHL xeno-transplant models after the first intravenous administration of PBS, free ADR, PC-ADR-BSA, and PC-ADR-Fab. Tumor volumes were measured every 3 days. Results are presented as mean ± SD of four separate mice in one group. →, treatment. **(D)***In vivo* antitumor therapeutic effects in disseminated human NHL xeno-transplant models after the first intravenous administration of PBS, free ADR, PC-ADR-BSA, and PC-ADR-Fab. Survival curves were plotted with the Kaplan-Meier method and were compared by using a log-rank test.

In order for *in vivo* distribution and tumor accumulation assays, lymphoma-bearing SCID mice were injected with free ADR and ADR-loaded liposomes (PC-ADR-BSA and PC-ADR-Fab) via tail vein. Twenty-four hours after treatment, tissues were harvested and the sum total ADR was extracted and measured. Figure 6A shows that there was a significant increase in tumor ADR accumulation in PC-ADR-Fab compared with PC-ADR-BSA (*p = 0.048) and free ADR-treated mice (**p = 0.000). The heart, liver, spleen, and lungs all showed less ADR accumulation with liposomal ADR treatment than with free ADR treatment. There was no difference in ADR accumulation among treatments for the kidneys. The displayed fluorescent image of different frozen sections (Figure 6B) also demonstrated distinct enhancement of red fluorescence in tumor tissues of mice treated with ADR-loaded liposomes compared with that treated with free ADR, and the administration of PC-ADR-Fab can induce more retention of ADR in tumor tissues than the administration of PC-ADR-BSA for the active targeting of Fab fragments.

### *In vivo* antitumor activity assessment

For the evaluation of *in vivo* antitumor activities, both the disseminated and localized human NHL xeno-transplant models were set up. In the localized model, Daudi cells were inoculated subcutaneously in the right flank of SCID mice. When the tumors reached about 50-60 mm^3^ in volume, mice were randomly treated with free ADR, PC-ADR-BSA and PC-ADR-Fab with an equivalent ADR amount of 5 mg/kg [25,38]. The mice were treated once a week for SCID mice based on previous study and our preliminary experimental results [39]. The tumor volume was recorded and illustrated in Figure 6C. Our results indicated that mice treated with PC-ADR-Fab and PC-ADR-BSA demonstrated a remarkable decrease in tumor burden compared with free ADR and control treatment as measured by tumor volume. Otherwise, PC-ADR-Fab exhibit a more excellent antitumor ability comparing with PC-ADR-BSA, with 2/4 mice of complete remission (CR) indicated by no measurable mass.

The excellent antitumor activity of our liposome is validated using a disseminated model, in which Daudi cells were transplanted intravenously into SCID mice via tail vein. After 48 h, these mice were randomly administered injections of PBS, free ADR, PC-ADR-BSA, and PC-ADR-Fab for three times once a week. Survival curves were plotted with the Kaplan-Meier method and were compared by using a log-rank test [33,34]. As illustrated in Figure 6D, ADR-loaded liposome (PC-ADR-BSA and PC-ADR-Fab) treatment significantly prolonged the survival of tumor-bearing mice compared to free ADR and PBS control treatment (*p < 0.05). As our expectation, comparing with PC-ADR-BSA treatment, the administration of PC-ADR-Fab led to significant prolongation of graft survival days (*p < 0.05), with a CR percentage of 4/10 indicated by long-term survival (>120 days post-treatment).

## Discussion

NHL presents not only as a solid tumor of lymphoid cells in lymph nodes and/or extranodal lymphatic organs, but also as free lymphoma cells in circulating blood [1-3]. Unlike most other malignancies, chemotherapy but not surgery plays the most important role in curing NHL [4-6]. Currently, more and more studies are focusing on finding out novel drug delivery system for treating solid tumors [7,11,17,25]. However, for the elimination of free malignant cells in circulating blood, high serum stability and specificity to tumor cells are of great importance.

In this study, we have successfully fabricated a rituximab Fab-conjugated liposome based on PC, of which the well-defined spherical morphology was observed under TEM. Because PC is a kind of diacetylenic lipids, which can form intermolecular cross-linking through the diacetylenic group by UV irradiation to form chains of covalently linked lipids in the liposomal bilayers (Additional file 1: Figure S1) [26], this covalently union between lipid chains leads to a relatively more compact structure; thus, an important impact on the stability of the polymerized drug delivery system can be obtained. This enhanced serum stability can result in longer-time circulation and slower clearance of encapsulated drugs *in vivo*. Further experimental results revealed a favorable biological compatibility of the liposome. All the abovementioned properties are of vital importance for an ideal drug delivery system in eliminating malignant lymphoma cells, especially those in the peripheral blood.

In order to determine the antitumor activities, we took two lymphoma cell lines, Raji and Daudi, as study targets. The experimental results demonstrated that the *in vitro* cytotoxicity of ADR-loaded targeting liposome (PC-ADR-Fab) was significantly promoted due to the enhancement of drug uptaking compared with free drugs and ADR-loaded non-targeting liposome. The result may be ascribed to the following two reasons. Firstly, previous studies have proven that nanoparticles are taken up by cells via clathrin and/or caveoli-mediated endocytosis unlike small molecule drugs, which were taken up by passive diffusion [40,41]. Thus, most nanoparticles can obviously enhance the intracellular uptake of chemotherapeutic agents, which was confirmed by previous studies and recognized as an important advantage of nanosized drug delivery system [25,42,43]. Secondly, the intracellular uptake could be further improved by the Fab fragments of rituximab based on the active targeting strategy by antigen-antibody identification and combination.

*In vivo* experimental results indicated that the immunoliposomes are selectively accumulated in tumor tissues, while the administration of free drugs resulted in high concentration of ADR in either normal or malignant tissues with no specificity. This remarkable discrepancy can significantly improve the bioavailability and reduce the detrimental cytotoxicity of chemotherapeutic agents. The *in vivo* antitumor experiments carried out both in the localized and disseminated human NHL xeno-transplant models suggest that our immunoliposome was significantly more effective than either free ADR or non-targeting liposomal ADR in inhibiting primary tumor growth and prolonging the graft survival. What's more, our immunoliposome still showed great advantage in tumor suppressing efficacy when compared with other drug delivery systems. For example, comparing with the anti-CD30 antibody-conjugated liposomal doxorubicin constructed by *Ommoleila Molavi* et al., the treatment of which can respectively decrease the tumor burden to approximately 1/7 and approximately 1/2 in comparison with PBS and free ADR treatment [44]; our immunoliposome can remarkably decrease the tumor burden to approximately 1/14 and approximately 1/4, respectively. In our opinion, this exceptional excellent *in vivo* antilymphoma activity of the ADR-loaded Fab fragment-decorated liposome is the cooperative action of the following effects: (1) enhanced intracellular uptake due to effective endocytosis based on well-defined liposomal structure and size distribution; (2) enhanced serum stability and controlled drug release (as a result of UV irradiation polymerizing) can contribute to long circulation time and durable antilymphoma activity; (3) enhanced tumor accumulation and retention *in vivo* through dual targeting function, passive targeting through EPR effects and active targeting through antigen-antibody reaction.

## Conclusions

In this study, we have identified a novel liposomal drug delivery system, PC-Fab, for improved chemotherapy of CD20-positive NHL. The *in vitro* and *in vivo* experimental results clearly suggested that this Fab fragment-decorated liposome can be a promising weapon in combating NHL, which deserves further investigation for clinical application.

## Competing interests

The authors declare that they have no competing interests.

## Authors’ contributions

CW, HL, and AD designed the experimental scheme; HL and HZ performed the preparation and characterization of the liposomes. HL, HZ, WZ, YC, ZY, QL, YW, and XT participated in the *in vitro* and *in vivo* cytotoxicity assay; HL drafted the manuscript; and CW and AD modified the manuscript. All authors read and approved the final manuscript.

## Supplementary Material

Additional file 1: Figure S1 Formulation and schematic diagram. Formulation and schematic diagram of irrad and non-irrad liposomes. Click here for file

Additional file 2: Figure S2 Time-concentration curve. Time-concentration curve of free and liposomal ADR by PK software. Click here for file
