# Supplementary figures and images for: Potentiating antilymphoma efficacy of chemotherapy using a liposome for integration of CD20 targeting, ultra-violet irradiation polymerizing, and controlled drug delivery
Source: Nanoscale Res Lett. 2014 Aug 28;9(1):447. doi: 10.1186/1556-276X-9-447 (PMC4151082; doi:10.1186/1556-276X-9-447)

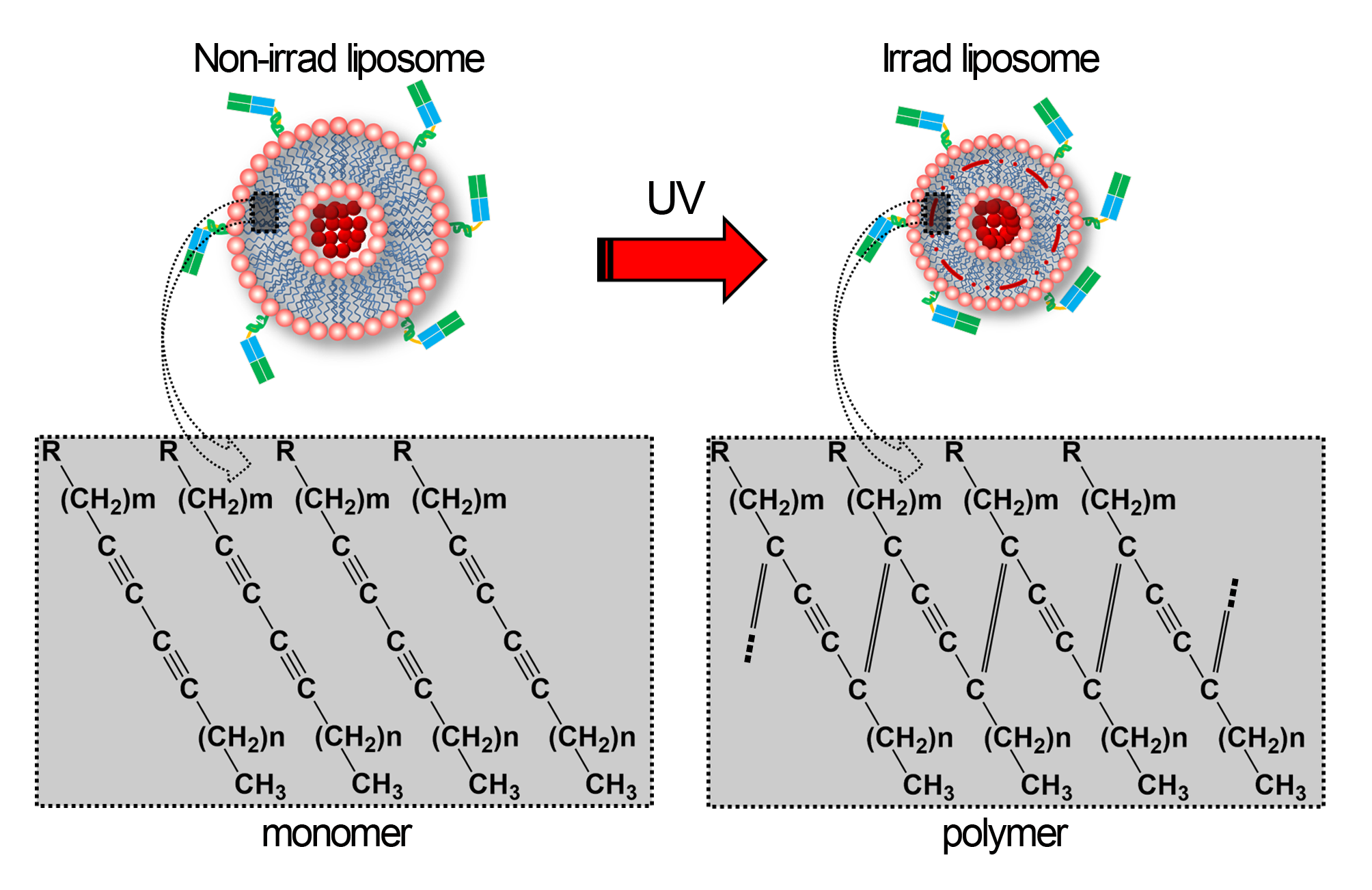

Supplement: Additional file 1: Figure S1 — Formulation and schematic diagram. Formulation and schematic diagram of irrad and non-irrad liposomes. [file 1556-276X-9-447-S1.tiff]
